# Prediction of resection after preoperative FOLFIRINOX in patients with localized pancreatic adenocarcinoma: a Trans-Atlantic Pancreatic Surgery (TAPS) Consortium study

**DOI:** 10.1093/jnci/djag033

**Published:** 2026-05-09

**Authors:** Eva M M Verkolf, Jacob L van Dam, Esther N Dekker, Quisette P Janssen, Laura R Prakash, Anissa DeSilva, Roeland F de Wilde, Marc G Besselink, Alice C Wei, Amer H Zureikat, Matthew H G Katz, Ching-Wei D Tzeng, Bas Groot Koerkamp, Trans-Atlantic Pancreatic Surgery (TAPS) Consortium, Eva M M Verkolf, Jacob L van Dam, Esther N Dekker, Quisette P Janssen, Laura R Prakash, Anissa DeSilva, Roeland F de Wilde, Marc G Besselink, Alice C Wei, Amer H Zureikat, Matthew H G Katz, Ching-Wei D Tzeng, Bas Groot Koerkamp

**Affiliations:** Department of Surgery, Erasmus MC Cancer Institute, Rotterdam, the Netherlands; Department of Surgery, Erasmus MC Cancer Institute, Rotterdam, the Netherlands; Department of Surgery, Erasmus MC Cancer Institute, Rotterdam, the Netherlands; Department of Surgery, Erasmus MC Cancer Institute, Rotterdam, the Netherlands; Department of Surgical Oncology, Division of Surgery, The University of Texas MD Anderson Cancer Center, Houston, TX, United States; Division of Surgical Oncology, University of Pittsburgh Medical Center, Pittsburgh, PA, United States; Department of Surgery, Erasmus MC Cancer Institute, Rotterdam, the Netherlands; Department of Surgery, Amsterdam UMC, University of Amsterdam, Amsterdam, the Netherlands; Department of Surgical Oncology, Cancer Center Amsterdam, Amsterdam, the Netherlands; Department of Surgery, Memorial Sloan Kettering Cancer Center, New York, NY, United States; Division of Surgical Oncology, University of Pittsburgh Medical Center, Pittsburgh, PA, United States; Department of Surgical Oncology, Division of Surgery, The University of Texas MD Anderson Cancer Center, Houston, TX, United States; Department of Surgical Oncology, Division of Surgery, The University of Texas MD Anderson Cancer Center, Houston, TX, United States; Department of Surgery, Erasmus MC Cancer Institute, Rotterdam, the Netherlands

## Abstract

**Background:**

Neoadjuvant chemotherapy is increasingly used for patients with localized pancreatic ductal adenocarcinoma (PDAC), although the likelihood of subsequent resection remains uncertain. The aim of this study was to develop a prediction model using baseline characteristics to estimate the probability of meeting prespecified criteria for resection after induction FOLFIRINOX.

**Methods:**

This retrospective study included patients with localized PDAC who received (m)FOLFIRINOX as initial treatment in 5 referral centers in the United States and the Netherlands (2012-2019). Multivariable logistic regression identified independent predictors of resection and was used to develop a prediction model.

**Results:**

Among 1835 patients, 18.9% were classified as potentially resectable (PR), 28.9% as borderline resectable (BR), and 52.2% as locally advanced (LA). Observed resection rates were 70.5% for PR, 53.1% for BR, and 17.8% for LA PDAC. Unfavorable independent factors for resection were advanced anatomical stage (BR, OR = 0.51; 95% CI = 0.37 to 0.69 and LA, OR = 0.11; 95% CI = 0.08 to 0.15), baseline CA19-9 >500 U/mL (OR = 0.60; 95% CI = 0.46 to 0.77), a WHO performance status of ≥1 (OR = 0.41; 95% CI = 0.33 to 0.52), and tumor size on baseline imaging >40 mm (OR = 0.62; 95% CI = 0.48 to 0.80). Depending on these factors, the predicted probability of resection ranged from 6.7% to 81.7%.

**Conclusion:**

At diagnosis, the probability of resection after induction (m)FOLFIRINOX for localized PDAC ranged from 7% to 82%, depending on anatomical stage, CA19-9 level, performance status, and tumor size at baseline. This prediction model may help communicate realistic expectations for the probability of resection.

## Introduction

Pancreatic ductal adenocarcinoma (PDAC) is the third leading cause of cancer-related mortality in Western countries.^1^ Patients with localized PDAC are classified based on imaging as potentially resectable (PR), borderline resectable (BR), and locally advanced (LA) PDAC.^2^^,^^3^ For patients with BR and LA PDAC, neoadjuvant or induction chemotherapy is the standard of care.^2^ For patients with PR PDAC, ongoing randomized controlled trials (RCTs) compare upfront surgery followed by adjuvant therapy vs neoadjuvant chemotherapy followed by surgery.^2^^,^^4-10^ Outside clinical trials, patients with PR PDAC are increasingly treated with systemic chemotherapy first.

Many patients with localized PDAC may benefit from surgical resection after systemic chemotherapy. Whether to perform a resection requires thoughtful shared decision-making considering surgical risk, oncological benefit (improved survival and/or quality of life), and individual patient preferences.^11^ This shared decision-making considers baseline patient and tumor characteristics, as well as restaging after systemic chemotherapy including disease progression on imaging, carbohydrate antigen 19-9 (CA19-9) change, and patient performance status.

At the time of diagnosis, most patients with localized PDAC are highly focused on the prospect of future resection, often viewing it as a primary treatment goal.^12^ This perspective is driven by the assumption that resection of localized disease results in cure.^13^ Unfortunately, most patients with localized PDAC on imaging have occult metastatic disease at diagnosis, which is reflected by a high rate of recurrence after resection and a 10-year overall survival of less than 5%.^14^ Adequate counseling includes explaining that surgical resection is not always in the patient’s best interest. The shared decision to perform a resection requires information that becomes available only at restaging. Nevertheless, patients could benefit from setting realistic expectations at diagnosis about the probability that surgical resection after systemic treatment will benefit them.

Several studies have reported the probability of resection after systemic chemotherapy for PR, BR, and/or LA PDAC. No large studies have investigated additional baseline patient and tumor characteristics to predict resection after systemic chemotherapy. The aim of this study was to develop a prediction model using baseline patient and tumor characteristics to estimate the probability of resection after initial FOLFIRINOX for patients with localized PDAC treated in tertiary referral centers.

## Methods

### Study design

This was a retrospective cohort study from the Trans-Atlantic Pancreatic Surgery (TAPS) Consortium, which created a real-world database of consecutive patients from high-volume pancreatic referral centers. The consortium included centers from the United States (The University of Texas MD Anderson Cancer Center, Memorial Sloan Kettering Cancer Center, and The University of Pittsburgh Medical Center) and the Netherlands (Amsterdam UMC and Erasmus MC Cancer Institute).^15^^,^^16^ All centers received ethical approval from local institutional review boards and legal approval for data sharing of deidentified data. A waiver of informed consent was obtained due to the retrospective nature of the study. This study followed the Strengthening the Reporting of Observational Studies in Epidemiology (STROBE) guidelines.

### Patients

The TAPS consortium has composed a cohort of consecutive patients with localized PDAC who received at least 1 cycle of (m)FOLFIRINOX as initial treatment between January 1, 2012 and December 31, 2019. Collected data included characteristics of the patient, tumor, and treatment, as well as clinical and pathological outcomes. All survival outcomes were last updated on October 22, 2022. After collection, data were deidentified and merged into a cloud-based digital research environment (Microsoft Azure DRE, Nijmegen, the Netherlands).

Baseline characteristics included age, sex, BMI, WHO performance status, and Charlson comorbidity index. Levels of the tumor markers carcinoembryonic antigen (CEA) and CA19-9 were collected before the first cycle, preferably at the time of normalized bilirubin levels (ie, <1.2 m/dL). Tumor size on baseline imaging was measured in millimeters and classified using the 8th edition of the AJCC TNM classification.^17^ Staging at diagnosis into PR, BR, and LA PDAC was based on radiographic imaging. The MD Anderson Cancer Center (MDACC) used the MDACC Clinical Classification System.^18^ All other centers used the National Comprehensive Cancer Network (NCCN) criteria, which differs from the MDACC Classification based on venous contact of the tumor.^2^ According to the NCCN criteria a PR PDAC has a maximum of 180 degrees of venous contact without contour irregularity, whereas the MDACC classification considers a tumor with any degree of venous contact as PR PDAC if there is no occlusion and no arterial contact.

### Treatment

A regimen of (m)FOLFIRINOX in full dose consisted of 2400 mg/m^2^ fluorouracil, 400 mg/m^2^ leucovorin, 180 mg/m^2^ irinotecan, and 85 mg/m^2^ oxaliplatin with or without a bolus of 400 mg/m^2^ of 5-fluorouracil. The multidrug regimen was repeated every 2 weeks. If toxicity occurred, dose adjustments or changes were made. Some patients had a chemotherapy switch, mostly due to local progression or toxicity.

Patients were evaluated for surgical resection by a multidisciplinary team meeting after restaging. Imaging at restaging aimed to detect metastatic disease and compare tumor size and vascular involvement with baseline imaging. Treatment response was also evaluated by comparing CA19-9 at restaging with baseline CA19-9 after biliary drainage. Performance status and surgical risk were also reassessed. The final decision to proceed with resection was made collaboratively, incorporating patient preferences and aligning treatment goals with clinical feasibility and expected outcomes.

Pathological staging of the resected specimen was based on the 8th edition of the TNM classification manual by the American Joint Committee on Cancer Staging.^19^ The resection margin status was considered R0 when ≥1 mm of the resection or dissection margin was free of tumor.^20^

### Statistical analysis

Analyses were performed using R software, version 4.1. Continuous variables of the baseline characteristics were presented as medians with interquartile ranges, and categorical variables as frequencies with proportions. Comparison of the continuous variables was done using the Kruskal-Wallis rank sum test, and the χ^2^ test was used to compare categorical variables.

### Prediction model

A prediction model was developed and validated using an internal-external validation design with split-sample approach to evaluate the predictive value of factors identified through multivariable logistic regression analysis.^21^ The cohort was initially divided based on year of diagnosis into a development cohort (2012-2016) and a validation cohort (2017-2019). The patients with missing data required for the model were excluded from the cohorts. A multivariable logistic regression model was created using the development cohort, incorporating variables with a *P*-value of below .20 identified in the univariable analysis. Internal validation was performed via bootstrapping with 200 resample subsets to assess model robustness. Temporal validation was performed by applying the final model from the development cohort to the temporally distinct validation cohort, which originated from the same referral centers. Model performance was assessed using discrimination and calibration metrics. After validation, a final model was refitted using the entire dataset (2012-2019) to maximize statistical power. Additionally, the multivariable logistic regression model was extended to include interaction terms between stage and the baseline predictors to evaluate potential effect modification. This final model was subsequently used to develop an online calculator that estimates the likelihood of resection for an individual patient with localized PDAC based on pretreatment characteristics, which is available via www.pancreascalculator.com.

## Results

### Baseline characteristics

A total of 1835 patients who received initial treatment with (m)FOLFIRINOX for localized PDAC were included. The baseline characteristics are detailed in Table 1. Most patients were male (54.6%) with a median age at diagnosis of 64 years (IQR = 57-69), a WHO performance status ≥1 (60.7%), and a BMI of >25 (57.9%). Anatomical stage at diagnosis was PR in 346 patients (18.9%), BR in 531 patients (28.9%), and LA in 958 patients (52.2%). The median level of CA19-9 at baseline was 208 U/mL (IQR = 46-774). Most patients had a clinical T-stage of cT1-2 (60.3%).

**Table 1.** Baseline characteristics, treatment characteristics, and pathological outcomes by resection status.

Baseline characteristics	Overall (*n* = 1835)^a^	No resection (*n* = 1138)^a^	Resection (*n* = 697)^a^	*P* ^b^
Age, years, median (IQR)	64 (57-69)	64 (57-69)	63 (57-69)	.40
Sex, female, No. (%)	833 (45.4)	529 (46.5)	304 (43.6)	.20
Tumor stage, No. (%)				<.001
PR	346 (18.9)	102 (9.0)	244 (70.5)	
BR	531 (28.9)	249 (21.9)	282 (53.1)	
LA	958 (52.2)	787 (69.2)	171 (17.8)	
WHO performance status, No. (%)				<.001
0	718 (39.3)	342 (30.2)	376 (54.2)	
1	1036 (56.7)	737 (65.0)	299 (43.1)	
2–3	74 (4.0)	55 (4.8)	19 (2.7)	
BMI, kg/m^2^, No. (%)				.004
<18.5	52 (2.9)	42 (3.7)	10 (1.4)	
18.5–25	711 (39.2)	455 (40.4)	256 (37.1)	
25–30	665 (36.6)	387 (34.4)	278 (40.3)	
>30	387 (21.3)	241 (21.4)	146 (21.2)	
Tumor location, No. (%)				.006
Head/Uncinate	1223 (66.6)	730 (64.1)	493 (70.7)	
Neck/Proximal body	301 (16.4)	209 (18.4)	92 (13.2)	
Distal body/Tail	311 (16.9)	199 (17.5)	112 (16.1)	
Tumor size, No. (%)				<.001
≤20 mm (cT1)	67 (3.8)	28 (2.5)	39 (5.9)	
>20 and ≤40 mm (cT2)	1003 (56.5)	563 (50.8)	440 (66.1)	
>40 mm (cT3)	704 (39.7)	517 (46.7)	187 (28.1)	
CA19-9 (U/mL), median (IQR)	208 (46-774)	265 (5-908)	149 (35-522)	<.001
CA19-9 > 500 (U/mL), No. (%)	559 (32.5)	393 (36.8)	166 (25.4)	<.001
**Treatment characteristics**				
No. cycles, median (IQR)	6 (4-8)	6 (4-8)	6 (4-8)	.20
Chemotherapy switch, No. (%)	236 (12.9)	150 (13)	86 (12)	.037
Radiotherapy, No. (%)	888 (49.0)	621 (55.4)	267 (38.6)	<.001
Adjuvant chemotherapy, No. (%)	413 (59.3)	–	413 (59.3)	
**Pathological outcomes**				
Tumor grade, No. (%)				–
Well	–	–	21 (3.3)	
Moderate	–	–	402 (62.2)	
Poor	–	–	188 (29.1)	
Tumor stage, No. (%)^c^				–
ypT0	–	–	33 (4.8)	
ypT1-2	–	–	493 (72.0)	
ypT3-4	–	–	154 (22.5)	
Nodal status, No. (%)^c^				–
ypN0	–	–	302 (44.2)	
ypN1	–	–	245 (35.8)	
ypN2	–	–	137 (20.0)	
R0 Resection, No. (%)	–	–	405 (66.1)	–
Lymphovascular invasion, No. (%)	–	–	370 (55.2)	–
Perineural invasion, No. (%)	–	–	512 (75.6)	–

Missing data: age (*n* = 1), WHO (*n* = 7), CA 19-9 (*n* = 113), BMI (*n* = 20), size (*n* = 61), cycles (*n* = 1), radiotherapy (*n* = 24), procedure (*n* = 3), margin (*n* = 84), differentiation (*n* = 86), ypT (*n* = 12), ypN (*n* = 13), perineural (*n* = 20), lymphovascular (*n* = 27), adjuvant (*n* = 1).

a Median (IQR); *n* (%).

b Wilcoxon rank sum test; Pearson’s χ^2^ test; Fisher exact test.

c According to the 8th edition of the AJCC TNM classification for pancreatic cancer.

Abbreviations: BR = borderline resectable; CA19-9 = carbohydrate antigen 19-9; LA = locally advanced; PR = potentially resectable.

### Treatment characteristics

The initial treatment of (m)FOLFIRINOX was administered with a median number of 6 cycles (IQR= 4-8). Only 128 patients (7.0%) received 2 cycles or fewer. A chemotherapy switch was performed in 236 patients (12.9%), because of local disease progression or toxicity. Subsequent radiotherapy was administered in 888 patients (49.0%). Surgical exploration with an intent of resection was performed in 841 patients (45.8%), of whom 697 patients (38.0% of entire cohort or 82.9% of patients taken to the operating room) underwent a resection. Reasons for not performing surgical exploration included metastatic disease (19.2%), unresectable disease (27.8%), or clinical deterioration (4.9%; Table S1). The same reasons were observed for not undergoing a resection, with 8.9% due to occult metastatic disease, 7.7% due to unresectable disease, or other factors encountered during surgical exploration (eg, liver cirrhosis; <1%; Table S2). Additional, or adjuvant, chemotherapy was administered in 413 patients (59.3%).

### Pathological outcomes

The median tumor size at pathological examination was 25 mm (IQR = 17-35). The resection margin status was R0 (ie, ≥1 mm) in most patients (66.1%), and tumor differentiation was mostly moderate to poor (91.3%). The pathological T-stage was ypT1-2 in 493 patients (72.0%), and ypT3-4 in 154 patients (22.5%). A complete pathological response (ie, ypT0) was found in 33 patients (4.8%). Most patients had a positive lymph node status (55.8%), perineural invasion (75.6%), and/or lymphovascular invasion (55.2%).

### Predictive factors for resection

Six baseline patient and tumor characteristics were predictive of resection with a *P*-value below .20 (Table 2). These factors included BMI, tumor stage (ie, PR, BR, or LA), baseline CA19-9, tumor location (ie, head, neck, or tail), and tumor size on baseline imaging. Only 4 of these factors were *independent* factors for the probability of resection: tumor stage, baseline CA19-9, WHO performance status, and tumor size on baseline imaging. After testing for interaction between stage (PR, BR, LA) and the independent factors for probability of resection included in our model, none of the interaction terms were statistically significant (global likelihood ratio test χ^2^ = 6.64, *df* = 6, *P* = .35; Table S3).

**Table 2.** Univariable analysis of baseline patient and tumor characteristics for the chance of resection.

	Univariable		
Characteristic	OR	95% CI	*P*
Age	0.99	0.98 to 1.01	.227
Sex, female	0.85	0.65 to 1.11	.226
BMI			
18.5–25	Ref		
<18.5	0.30	0.07 to 0.89	.056
25–30	1.50	1.10 to 1.24	.010
>30	0.86	0.59 to 1.24	.409
Tumor stage			
PR	Ref		
BR	0.52	0.33 to 0.79	.002
LA	0.13	0.08 to 0.19	<.001
CA19-9 >500 (U/mL)	0.79	0.58 to 1.06	.118
WHO ≥1	0.35	0.26 to 0.46	<.001
Tumor location			
Head/Uncinate	Ref		
Neck/Proximal body	0.71	0.49 to 1.03	.073
Distal body/Tail	0.87	0.59 to 1.26	.471
Tumor size			
≤40 mm (cT1-2)	Ref		
>40 mm (cT3-4)	0.47	0.35 to 0.64	<.001

Abbreviations: CI = confidence interval; BR = borderline resectable; CA19-9 = carbohydrate antigen 19-9; LA = locally advanced; PR = potentially resectable; OR = odds ratio.

### Prediction model for meeting criteria for surgical resection

**Figure 1.**
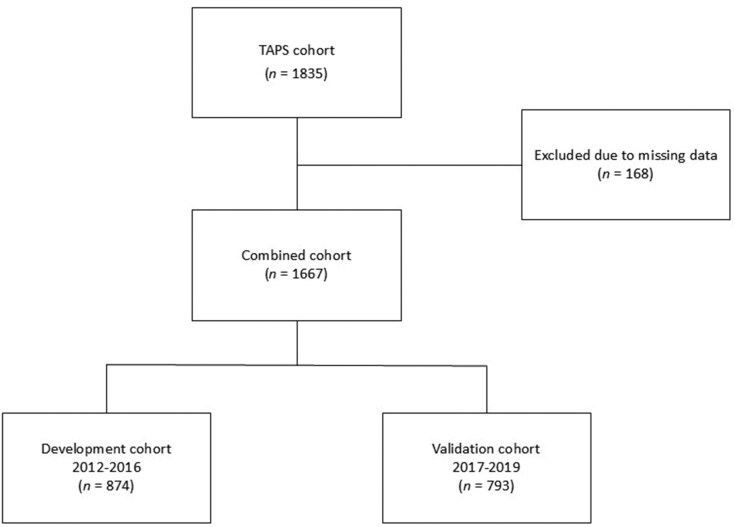
Flow diagram showing patient inclusion for development and validation cohorts.

**Figure 2.**
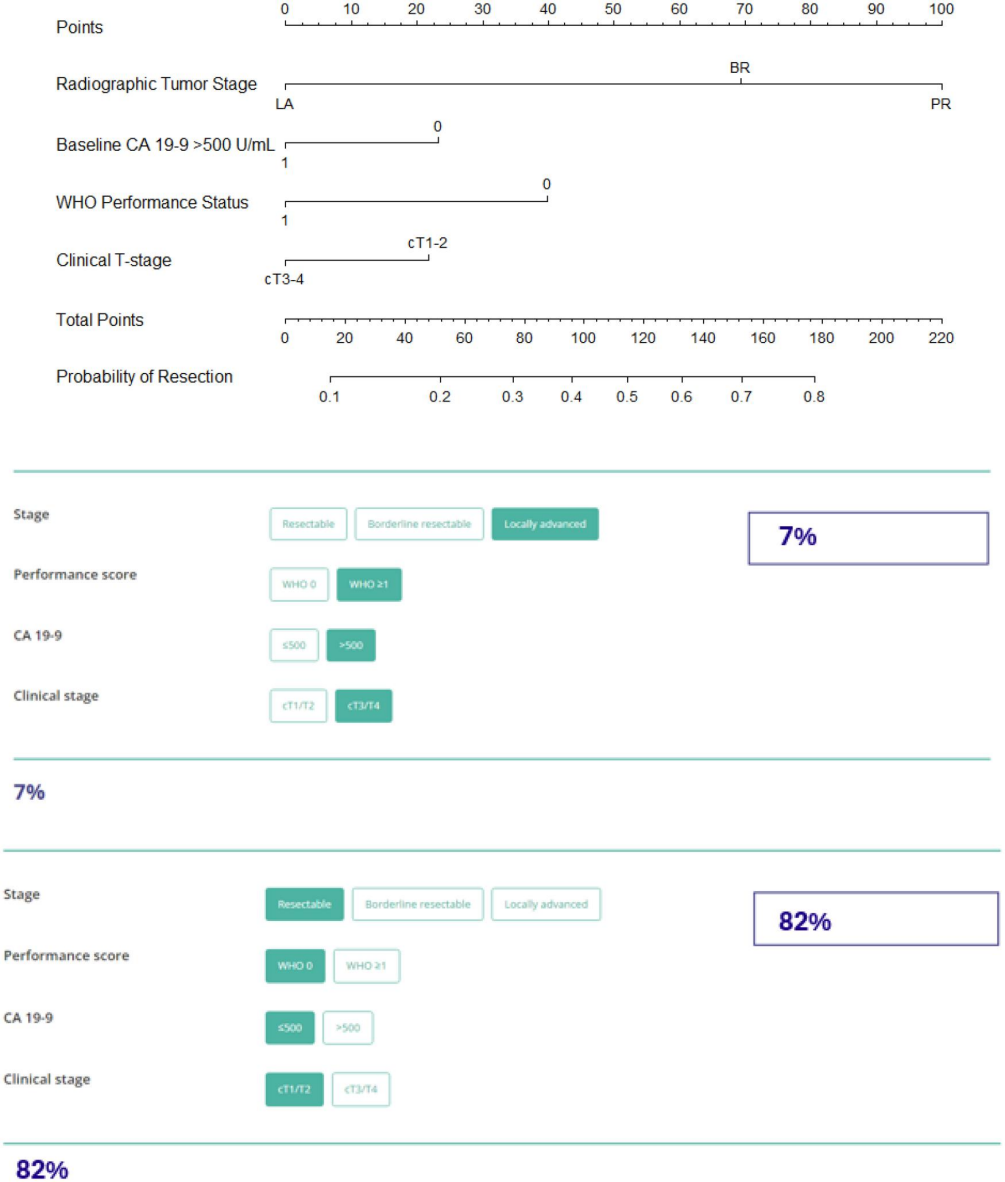
**A)** Nomogram for predicting the chance of resection in patients with localized pancreatic ductal adenocarcinoma. **B)** Online calculator.

After excluding 168 patients (9.2%) with missing data for one of the independent factors, a total of 1667 patients formed the combined cohort (Figure 1). The 874 patients in this cohort diagnosed between 2012 and 2016 formed the development cohort, which was used to develop the multivariable logistic regression model. The model incorporated all 4 independent prognostic factors. The validation cohort, consisting of 793 patients diagnosed between 2017 and 2019, was employed for external validation. In the combined cohort, BR stage was associated with a lower probability of resection (OR = 0.51, 95% CI = 0.37 to 0.69), and LA stage further decreased the probability of resection (OR = 0.11, 95% CI = 0.08 to 0.15). A baseline CA19-9 level >500 U/mL was also predictive of reduced resection rates (OR = 0.60, 95% CI = 0.46 to 0.77), as was a WHO performance status of ≥1 (OR = 0.41, 95% CI = 0.33 to 0.52) and a clinical T-stage of cT3-4 (OR = 0.62, 95% CI = 0.48 to 0.80). The model demonstrated good discrimination, with area under the curve values ranging from 0.767 to 0.803 across the development, validation, and combined cohorts (Table 3). Calibration showed minimal deviation from ideal calibration. The prediction model is available as an online calculator on the pancreascalculator website (www.pancreascalculator.com). A nomogram visually represents the contribution of the 4 factors on the probability of resection (see Figure 2).

**Table 3.** Multivariable analysis of the development, validation, and combined cohort.

	Development cohort (*n* = 874)	Validation cohort (*n* = 793)	Combined cohort (*n* = 1667)
**Predictors—OR (95% CI)**			
Stage			
PR	1.00	1.00	1.00
BR	0.49 (0.30 to 0.78)	0.54 (0.35 to 0.81)	0.51 (0.37 to 0.69)
LA	0.13 (0.08 to 0.21)	0.09 (0.06 to 0.14)	0.11 (0.08 to 0.15)
CA19-9 >500 (U/mL)	0.73 (0.52 to 1.03)	0.49 (0.34 to 0.70)	0.60 (0.46 to 0.77)
WHO ≥1	0.35 (0.25 to 0.49)	0.50 (0.36 to 0.70)	0.41 (0.33 to 0.52)
Tumor size >40 mm (cT3)	0.55 (0.38 to 0.76)	0.71 (0.49 to 1.02)	0.62 (0.48 to 0.80)
**Model performance**			
*Discrimination—C-index*			
Area under the curve apparent	0.767 (0.730 to 0.799)	0.803 (0.769 to 0.832)	0.787 (0.763 to 0.809)
Area under the curve validated	0.769 (0.739 to 0.801)^a^	0.795 (0.762 to 0.825)^b^	0.787 (0.762 to 0.813)^a^
*Calibration*			
Intercept	0.00 (−0.16 to 0.16)	0.23 (0.07 to 0.39)^b^	0.00 (−0.11 to 0.11)
Slope	1.00 (0.84 to 1.16)	1.03 (0.87 to 1.19)^b^	1.00 (0.89 to 1.11)

a Estimated by internal validation using bootstrapping.

b Estimated by applying the model derived from the development cohort in the validation cohort.

Abbreviations: CI = confidence interval; BR = borderline resectable; CA19-9 = carbohydrate antigen 19-9; LA = locally advanced; PR = potentially resectable; OR = odds ratio.

The probability of undergoing a resection was 70.5% for PR, 53.1% for BR, and 17.8% for LA PDAC. By applying the prediction model, more individualized predictions of the probability of resection could be made. The predicted probability ranged from 6.7% to 81.7% when also taking CA19-9 baseline level, WHO performance status at baseline, and tumor size on baseline imaging into account. Patients with PR PDAC varied in the probability from 33.3% to 81.7%, patients with BR PDAC from 7.7% to 73.6%, and patients with LA PDAC from 6.7% to 31.0%.

## Discussion

This multicenter study resulted in a model to estimate the probability of undergoing a resection after neoadjuvant or induction (m)FOLFIRINOX using data available at the time of initial staging of patients with localized PDAC. The aim was to provide realistic expectations, at diagnosis, that surgery would become the preferred treatment after systemic chemotherapy. The model identified independent predictive factors decreasing the probability of resection, including advanced tumor stage (ie, BR or LA), worse biology with baseline CA19-9 above 500 U/mL, and a WHO performance status of 1 or higher.^22^ In addition to these “ABC” factors, larger tumor size on baseline imaging (eg, cT3-4) was an independent unfavorable predictor of the probability of resection after induction (m)FOLFIRINOX. Each of the 4 unfavorable factors (BR PDAC, CA19-9 > 500, WHO ≥1, or cT3-4) resulted in about a 2-fold reduction in the probability of ultimately undergoing resection. LA PDAC resulted in a 10-fold reduction in the probability of resection compared with patients with PR PDAC. Depending on the combination of these factors, the probability of being considered a candidate for resection ranged from 7% for patients with all unfavorable factors to 82% for patients without any unfavorable factors. The model is available via pancreascalculator.com.

Several studies have described the proportion of patients undergoing a resection after neoadjuvant treatment of (borderline) resectable PDAC. A recent meta-analysis of 7 RCTs compared neoadjuvant therapy with upfront surgery in patients with PR and BR PDAC.^4^ The pooled proportion of patients undergoing a resection after neoadjuvant chemotherapy was 72%, which is similar to the 70.5% of PR PDAC patients in our cohort who underwent resection. A meta-analysis of 313 patients with BR PDAC treated with FOLFIRINOX reported a proportion of resection of 67.8%.^23^ The included studies, however, were mostly small retrospective studies that differed in definitions for BR PDAC. The BR PDAC patients in our cohort had an overall probability of resection of 53.1%. When accounting for the independent predictive factors identified in our study, the probability of meeting criteria for resection for BR PDAC patients ranged from 7.7% to 73.6%, reflecting the spectrum of disease within the Borderline ABC classification.

The phase II RCT (JCOG 1407) compared (m)FOLFIRINOX vs nab-paclitaxel plus gemcitabine for 126 patients with LA PDAC.^24^ The probability of meeting criteria for surgical resection was low in both groups (8.1% vs 7.8%), compared with 17.8% in the present study. The probability was 25.9% in a meta-analysis of 12 cohort studies of 689 patients receiving FOLFIRINOX as initial treatment for LA PDAC.^25^ However, the included studies were small retrospective studies. Finally, the phase II NEOLAP trial randomly assigned 130 patients with LA PDAC to induction chemotherapy with nab-paclitaxel plus gemcitabine vs FOLFIRINOX after an initial 2 cycles of nab-paclitaxel plus gemcitabine for patients with LA PDAC.^26^ After induction chemotherapy, 23 patients (35.9%) in the nab-paclitaxel plus gemcitabine group and 29 patients (43.9%) in the FOLFIRINOX group underwent a surgical resection (*P* = .38). The high proportion in the NEOLAP trial, however, can be explained by the inclusion of patients only after stable disease at restaging after 2 cycles of induction chemotherapy.

The presented model used baseline patient and tumor characteristics to estimate the probability of surgical resection after initial (m)FOLFIRINOX for individual patients at the time of diagnosis. The actual shared decision whether to proceed with surgical exploration, however, is made after systemic treatment. This allows incorporating additional determinants such as changes in tumor size on imaging, changes in CA19-9 level, performance status after chemotherapy, and the occurrence of metastatic disease.

This is the first study that identified independent predictive factors of surgical resection after systemic treatment for patients with all stages of localized PDAC. However, this retrospective cohort study has a few inherent limitations. Anatomical criteria varied slightly between centers, because MDACC used their own criteria and the rest of the centers used the NCCN criteria.^2^ In addition, the NCCN criteria have been modified somewhat over time.^2^ Furthermore, participating centers differed in treatment approaches regarding chemotherapy switch and radiation. However, all centers are established pancreatic cancer referral centers, and the heterogeneity in treatment after FOLFIRINOX improved the generalizability of the study’s results. On the other hand, the results may be less applicable to patients not treated in experienced referral centers. Finally, the decision to perform a resection after initial (m)FOLFIRINOX was made by multidisciplinary teams together with the patients. This decision-making process involves nuances not available in a database. Criteria for this decision may differ between centers within and outside this consortium and continue to evolve over time.

The proposed prediction model incorporated anatomical classification, baseline CA19-9 level, patient performance status, and tumor size on baseline imaging. The resulting individualized probability of resection ranged from 7% to 82% for patients with localized PDAC who received (m)FOLFIRINOX as initial treatment.

## Supplementary Material

djag033_Supplementary_Data

## Data Availability

Data are available from the corresponding author upon reasonable request.
