# Supplementary material for: Prediction of resection after preoperative FOLFIRINOX in patients with localized pancreatic adenocarcinoma: a Trans-Atlantic Pancreatic Surgery (TAPS) Consortium study
Source: J Natl Cancer Inst. 2026 May 9;118(6):1089–96. doi: 10.1093/jnci/djag033 (PMC13247335; doi:10.1093/jnci/djag033)
Supplement: djag033_Supplementary_Data [file djag033_supplementary_data.docx]

**SUPPLEMENTARY MATERIAL**

**Supplementary Methods**

The supplementary material includes three additional tables. The first two tables provide detailed reasons why patients did not undergo surgical exploration and, among those explored, why resection was not performed. The third table presents results from an exploratory analysis in which the multivariable logistic regression model was extended to include interaction terms between stage (potentially resectable, borderline resectable, and locally advanced) and baseline predictors (serum CA19-9, WHO performance status, and tumor size) to assess potential effect modification.

**Table S1.** Reasons for not undergoing surgical exploration after initial treatment with FOLFIRINOX

| Evaluation | All patients | PR | BR | LA |
| --- | --- | --- | --- | --- |
| Total | 1835 | 346 | 531 | 958 |
| Proceed to surgery | 841 (45.8%) | 272 (78.6%) | 335 (63.1%) | 234 (24.4%) |
| No surgery b/o M+ on imaging | 353 (19.2%) | 39 (11.3%) | 93 (17.5%) | 221 (23.1%) |
| No surgery b/o unresectable disease on imaging | 511 (27.8%) | 15 (4.3%) | 71 (13.4%) | 425 (44.4%) |
| No surgical exploration b/o clinical deterioration | 90 (4.9%) | 10 (2.9%) | 24 (4.5%) | 56 (5.8%) |
| No surgical exploration b/o other reason | 5 (<0.3%) | 5 (1.4%) | 0 | 0 |
| Unknown | 35 (1.9%) | 5 (1.4%) | 8 (1.5%) | 22 (2.3%) |

Abbreviations: PR, potentially resectable; BR, borderline resectable; LA, locally advanced; b/o, because of; M+, metastatic disease.

**Table S2.** Reasons for not undergoing resection at surgical exploration

| Reason no resection at surgical exploration | Surgical cohort | PR | BR | LA |
| --- | --- | --- | --- | --- |
| Total | 841 | 272 | 335 | 234 |
| Resection | 697 (82.9%) | 244 (89.7%) | 282 (84.2%) | 171 (73.1%) |
| Locally advanced/unresectable | 65 (7.7%) | 4 (1.5%) | 28 (8.4%) | 33 (14.1%) |
| M+ | 75 (8.9%) | 22 (8.1%) | 23 (6.9%) | 30 (12.8%) |
| Other | 4 (<1%) | 2 (<1%) | 2 (<1%) | 0 |

Abbreviations: PR, potentially resectable; BR, borderline resectable; LA, locally advanced; M+, metastatic disease.

**Table S3.** Likelihood ratio tests for main effects and interactions between stage and baseline predictors (CA19-9, WHO performance status, tumor size) in relation to resection probability

| **Factor** | **Chi-square** | **Df** | **P-value** |
| --- | --- | --- | --- |
| **Main effects** | | | |
| Stage (PR/BR/LA) | 63.78 | 8 | <0.001 |
| CA19-9 >500U/mL | 15.04 | 3 | 0.002 |
| WHO PS ≥1 | 16.45 | 3 | 0.001 |
| cT3-4 vs cT1-2 | 2.99 | 3 | 0.394 |
| **Interaction terms** | | | |
| Stage x CA19-9 >500U/mL | 2.10 | 2 | 0.351 |
| Stage x WHO PS ≥1 | 2.80 | 2 | 0.247 |
| Stage x cT3-4 | 1.75 | 2 | 0.416 |
| **Global interaction (all stage x predictor)** | 6.64 | 6 | 0.355 |
| **Model LR (overall)** | 114.05 | 11 | <0.001 |

Abbreviations: Df, degrees of freedom; PR, potentially resectable; BR, borderline resectable; LA, locally advanced; CA19-9, carbohydrate antigen 19-9; WHO PS, World Health Organization performance status; cT1-4, clinical T-stage; LR, likelihood ratio.
